# Efficacy and Safety of Minocycline-Containing Bismuth Quadruple Therapies Versus Standard First-Line Bismuth Quadruple Therapies for *Helicobacter pylori* Eradication: A Systematic Review and Meta-Analysis

**DOI:** 10.3390/idr18010016

**Published:** 2026-02-06

**Authors:** Hakim Ullah Wazir, Abdul Muqeet Khuram, I M Khalid Reza, Hafsa Ajmal, Hafsa Parveen, Zeeshan Ahmed, Yousra Iftequar, Noora Inam, Ilyas Muhammad Sulaiman, Nayanika Tummala, Hafiz Muhammad Moaaz Sajid, Anum Zia Khan, Ussama Shafaqat

**Affiliations:** 1 Gajju Khan Medical College, Swabi 23561, Pakistan; hakim.wazir@gkmcs.edu.pk (H.U.W.); noorainam876@gmail.com (N.I.); ilyasms9464839@gmail.com (I.M.S.); 2 Department of Internal Medicine, University of Connecticut Health, Farmington, CT 06030, USA; 3 Department of Medicine, Enam Medical College and Hospital, Dhaka 1340, Bangladesh; khalidreza.emc@gmail.com; 4 Department of Medicine, Mayo Hospital, Lahore 54000, Pakistan; hafsaajmal120@gmail.com; 5 Department of Medicine, University of Louisville, Louisville, KY 40202, USA; h0parv01@louisville.edu; 6 Department of Medicine, King Edward Medical University, Lahore 06117, Pakistan; zeeshanahmed4214@gmail.com (Z.A.); ussama.shafaqat283@gmail.com (U.S.); 7 Department of Medicine, VRK Women’s Medical College, Hyderabad 500075, India; dr.yousraiftequar@gmail.com; 8 Department of Medicine, New York Medical College ST Marys St. Clares, Denville, NJ 07834, USA; nayantummala@gmail.com; 9 Punjab Medical College, Faisalabad Medical University, Faisalabad 38800, Pakistan; dr.m.moaazsajid@gmail.com; 10 Department of Medicine, Jinnah Sindh Medical University, Karachi 75510, Pakistan; anumzia741@gmail.com

**Keywords:** *H. pylori* eradication, helicobacter pylori infection, minocycline, bismuth quadruple therapy

## Abstract

Background: Growing antibiotic resistance and the limited availability of key components in standard Helicobacter pylori treatments have driven the search for effective alternatives. Minocycline, with its broad-spectrum activity and favorable pharmacokinetics, has emerged as a promising substitute. This meta-analysis compares the safety and efficacy of minocycline-containing bismuth quadruple therapy (MBQT) to conventional first-line BQT regimens, incorporating data from the recent study by Lin et al. Methods: The inclusion criteria were randomized controlled trials (RCTs) with a target population of both treatment-naïve and previously treated patients diagnosed with Helicobacter pylori (*H. pylori*) infection. The intervention received by eligible patients was a minocycline–bismuth quadruple therapy (MBQT) regimen containing bismuth, minocycline, proton pump inhibitors (PPI), and any additional antibiotic with a minimum period of 2 weeks of administration. We excluded study designs other than RCT and clinical trials that include patients without confirmed *H. pylori* infection, animal populations, in vitro experiments, and reports of other outcomes that did not include a minimum intervention duration of 2 weeks. A comprehensive literature search was conducted on PubMed, EMBASE, Cochrane Library, and ScienceDirect from inception to 20 May 2025. After screening via Rayyan, data were extracted on an Excel spreadsheet. Quality was assessed using the Cochrane RoB 2.0 tool. Eligible randomized controlled trials (RCTs) were included and analyzed using RevMan 5.4. Outcomes assessed were intention-to-treat and per-protocol eradication rates. Adverse effects were compared among therapies. A random-effects model was used; an I^2^ < 50% and *p*-value < 0.05 indicated homogeneity and significant results respectively. Results: Five RCTs with 7 interventions involving 2812 patients were included. The pooled odds ratio (OR) for MBQT in intention-to-treat (ITT) analysis was 1.25 (95% CI: 0.96–1.61), showing a non-significant trend. No heterogeneity was detected (I^2^ = 0.0%). In the modified ITT (mITT) analysis (2 studies), MBQT showed higher eradication (OR: 1.70, 95% CI: 0.00–1042.90), but wide CI and high heterogeneity (I^2^ = 70.7%) limited interpretation. All studies were included in the per-protocol (PP) analysis, which showed a statistically significant improvement with MBQT (OR: 1.67, 95% CI: 1.14–2.45) and low heterogeneity (I^2^ = 5.2%), suggesting consistent results. Although not statistically significant, MBQT was associated with a slightly lower rate of adverse events compared to standard therapy (OR: 0.81, 95% CI: 0.59–1.12). I^2^ = 50.6% showed moderate heterogeneity in safety outcomes. Discussion: the number of included RCTs was modest, with only five studies meeting eligibility criteria, and only two contributing to the modified intention-to-treat analysis. The risk-of-bias assessment showed variation in methodological quality across the included studies. Several studies exhibited high risk judgments in critical domains. particularly randomization, deviations from intervention, and selective reporting. Patients who completed the treatment benefited more from MBQT, which also had a comparable safety profile to conventional BQT regimens. In the treatment of *H. pylori* infection, MBQT may be considered a safe alternative for first-line treatment.

## 1. Introduction

Helicobacter pylori (*H. pylori*) is one of the most common chronic bacterial infections, colonizing the stomach of approximately 50% of the global population [1]. Its clinical significance stems from its strong association with major gastrointestinal diseases, including chronic gastritis, peptic ulcer disease, gastric adenocarcinoma, and gastric mucosa-associated lymphoid tissue (MALT) lymphoma [2]. In 1994, the International Agency for Research on Cancer classified *H. pylori* as a Group I carcinogen, reflecting its causal role in gastric malignancy [3]. Successful eradication of the organism not only heals peptic ulcers but also reduces recurrence, lowers the risk of gastric cancer, and improves quality of life, making eradication therapy an essential component of global gastric cancer prevention strategies [4].

Over the past two decades, rising antibiotic resistance has posed a major challenge to effective eradication therapy. Clarithromycin resistance, in particular, has reached alarming levels in many regions, exceeding 20–30% in parts of Asia, Southern Europe, and Africa [5]. Resistance to metronidazole is also widespread, further compromising the efficacy of traditional triple therapy regimens. Consequently, current international guidelines, including the Maastricht VI/Florence consensus and the American College of Gastroenterology recommendations, endorse bismuth quadruple therapy (BQT), comprising a proton pump inhibitor (PPI), bismuth, tetracycline, and metronidazole, as the preferred first-line treatment in areas with high clarithromycin resistance or in patients with penicillin allergy [6,7]. Despite its effectiveness, conventional BQT faces important barriers to widespread adoption. Tetracycline, a cornerstone of the regimen, is frequently unavailable in many countries due to limited production and distribution. Minocycline, on the other hand, is still widely accessible in many low- and middle-income nations and is frequently used to treat many inflammatory and infectious diseases [8]. Even when accessible, tetracycline’s four-times-daily dosing schedule is inconvenient, and gastrointestinal side effects such as nausea and abdominal discomfort often reduce adherence [9]. These practical limitations have led researchers to explore alternatives that could improve both patient compliance and therapeutic outcomes.

Minocycline, a semisynthetic tetracycline derivative, has emerged as a promising substitute. Compared with tetracycline, minocycline demonstrates higher lipid solubility, improved oral bioavailability, and a longer elimination half-life, allowing for twice-daily dosing [10]. Importantly, the resistance of *H. pylori* to minocycline remains rare, even in regions with significant resistance to other antibiotics [11]. Clinical trials and real-world studies have evaluated minocycline-containing bismuth quadruple therapy (MBQT) in both treatment-naive and treatment-experienced patients. Randomized controlled trials have demonstrated eradication rates exceeding 85% in intention-to-treat analyses, with per-protocol rates often approaching or surpassing 90% [12,13]. Furthermore, minocycline-based regimens have shown comparable safety profiles to conventional BQT, with the most notable adverse effect being dizziness, which is generally mild and transient Other adverse effects include abdominal discomfort and abdominal pain, diarrhea and rash [14]. Beyond tetracycline, modern BQT protocols have integrated various antibiotics, such as amoxicillin, clarithromycin, metronidazole, doxycycline, and cefuroxime. Clarithromycin resistance is extremely common worldwide and significantly decreases eradication success when used empirically. Metronidazole resistance is also common, necessitating greater doses or longer treatment periods, which increases the risk of side effects. Amoxicillin-containing regimens are not appropriate for people with penicillin allergies, whilst cephalosporins and doxycycline have minimal supporting evidence and varied efficacy across studies [15]. Recent systematic reviews and meta-analyses support the non-inferiority of MBQT compared with traditional tetracycline-based regimens. Pooled eradication rates have consistently exceeded 80% in intention-to-treat analyses and reached 90% in per-protocol populations, with compliance rates above 90% [16]. These findings suggest that minocycline is not only a feasible alternative in settings where tetracycline is unavailable but may also offer pharmacological and adherence advantages. Effective acid suppression, in addition to antibiotic selection, is a crucial factor in the effectiveness of *H. pylori* eradication. While more recent potassium-competitive acid blockers (P-CABs) may offer faster and long-lasting acid suppression, proton pump inhibitors (PPIs) improve antibiotic stability and efficacy by raising intragastric pH. Treatment outcomes may be further impacted by variations in the kind and strength of acid-suppressive medication used in different BQT regimens [17]. Given the global burden of *H. pylori* infection, the rising tide of antibiotic resistance, and the practical limitations of conventional BQT, it is essential to critically review the role of minocycline-containing regimens. This systematic review and meta-analysis, therefore, aims to compare the pooled efficacy, safety, and compliance of MBQT versus traditional tetracycline-based BQT as first-line treatments for *H. pylori* eradication in order to clarify whether minocycline can be considered a viable and evidence-based alternative in routine clinical practice.

## 2. Materials and Methods

This systematic review and meta-analysis were conducted according to the Preferred Reporting Items for Systematic Reviews and Meta-Analyses (PRISMA) criteria (Supplementary Materials) and registered with PROSPERO: CRD420251065240 (Date: 1 June 2025).

### 2.1. Data Sources and Search Strategy

This meta-analysis, entitled Efficacy and Safety of Minocycline-Containing Bismuth Quadruple Therapy Versus Standard First-Line Bismuth Quadruple Regimens for Helicobacter pylori Eradication, was conducted in accordance with Preferred Reporting Items for Systematic Reviews and Meta-Analysis (PRISMA) guidelines [18]. A comprehensive electronic search was conducted across PubMed (Medline), Embase, Scopus, Cochrane Central, and ScienceDirect databases from inception to May 2025. A detailed search strategy for the databases is provided in Appendix A.

### 2.2. Study Selection

**Figure 1:**
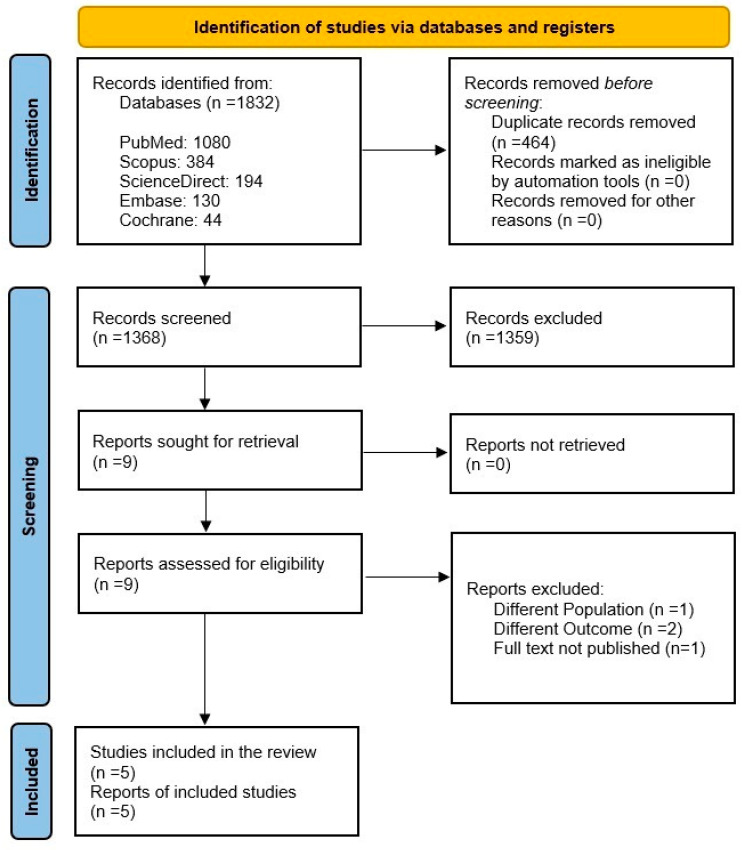
PRISMA Flowchart.

The inclusion criteria were randomized controlled trials (RCTs) with a target population of both treatment-naïve and previously treated patients diagnosed with Helicobacter pylori (*H. pylori*) infection. The intervention received by eligible patients was a minocycline-bismuth quadruple therapy (MBQT) regimen containing bismuth, minocycline, proton pump inhibitors (PPI), and any additional antibiotic with a minimum period of 2 weeks of administration. The diagnosed infection was confirmed through a urea breath test (UBT), histology, or rapid urease test (RUT). The comparator group received a bismuth quadruple therapy (BQT) regimen containing tetracycline, amoxicillin, or clarithromycin combined with bismuth and PPI. Figure 1 reveals identification of studies and databases.

We excluded narrative reviews, systematic reviews, meta-analyses, uncontrolled trials, case series, case reports, letters to the editor, and commentaries. Clinical trials including patients without confirmed *H. pylori* infection, animal populations, in vitro experiments, and reports of other outcomes that did not include a minimum intervention duration of 2 weeks were excluded. Observational studies, such as case–control, retrospective, or prospective cohort, and cross-sectional studies, were excluded. Articles such as peer-reviewed commentaries, letters to the editor, and case reports were also excluded. Additionally, we also excluded one RCT that compared minocycline-based BQT with a regimen other than conventional bismuth-based quadruple therapy. Since our review focused strictly on studies using conventional bismuth BQT as the comparator, this trial did not meet our inclusion criteria [19].

Authors independently screened titles and abstracts, followed by full-text assessments to identify the eligible studies. Duplicates were removed from the list after exporting the retrieved articles to Zotero Reference Library Software (Version 7, Zotero, Vienna, Virginia). All reviewers then carefully assessed the remaining articles, and only those that met the previously stated eligibility conditions were included. Any disagreements during screening were resolved through mutual consensus.

### 2.3. Data Extraction

Then reviewers extracted the data from the full-text assessment using an online Excel spreadsheet. The data collected from captured baseline study characteristics included study title, study ID, author, journal name, year of publication, design type, country of origin, sample size, minocycline-containing regimen, and non-minocycline, or another regimen. Baseline parameters were as follows: number of participants, mean age, sex distribution, body mass index (BMI), peptic ulcer disease, history of smoking, and history of alcohol consumption. Table 1 contains all the baseline characteristics for the included studies.

The primary outcomes were efficacy and safety, or the incidence of adverse events. Efficacy outcomes include intention-to-treat (ITT), per-protocol (PP), and modified intention-to-treat (mITT) eradication rates. Incidence of adverse events or safety outcomes was abdominal discomfort, nausea and vomiting, dizziness, taste distortion, diarrhea, anorexia, fatigue, constipation, total adverse events, and other adverse events, including skin rash, headache, insomnia, and darkened stool.

### 2.4. Quality Assessment

**Figure 2:**
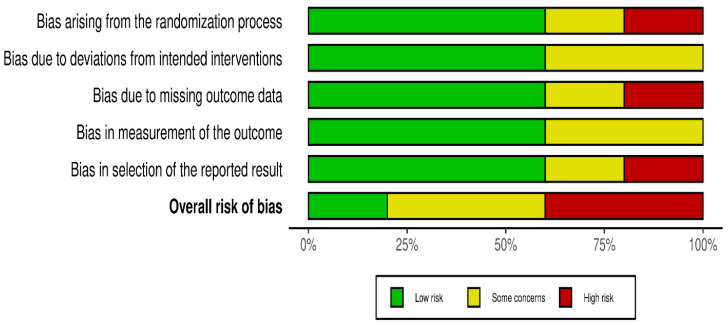
Risk of bias.

**Figure 3:**
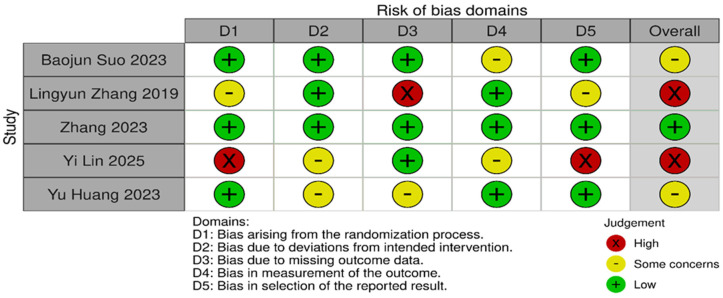
Risk of bias domains [13,20,21,22,23].

Risk of bias was assessed independently using the Cochrane Risk of Bias 2.0 tool. Quality assessment and risk of bias were independently assessed using the Cochrane Risk of Bias Tool 2.0 on all included RCTs. The assessment examines five key domains where bias might be introduced. These domains include bias arising from the randomization process, bias due to deviations from intended interventions, bias due to mixing outcome data, bias in the measurement of outcome data, and bias in the selection of reported results. Each domain was carefully evaluated and rated as having either a low risk of bias, some concerns, or a high risk of bias. Based on these individual assessments, we then assigned an overall risk of bias judgment for each RCT to guide the interpretation of study reliability. The quality assessment was performed by two independent investigators, and the results were matched. Any disagreements were resolved through discussions. A summary is shown in Figure 2 and Figure 3.

## 3. Results

### 3.1. Search Results

The comprehensive literature search identified 1832 studies After removing 464 duplicates, 1368 articles were screened on Rayyan. Finally, 5 RCTs were included in this systematic review and meta analysis. The details of the screening procedure are shown in Figure 1.

### 3.2. Study Characteristics

This meta-analysis included five randomized controlled trials (RCTs) with seven interventions and 2812 participants to compare the efficacy and safety of Minocycline-containing bismuth quadruple therapy (MBQT) to typical first-line bismuth quadruple therapies in Helicobacter pylori treatment. The baseline characteristics of the patients included are provided in Table 1. There was a total of 986 patients in the MBQT group and 986 in the standard therapy group.

### 3.3. Risk of Bias in Included Studies

Statistical heterogeneity was assessed using Cochran’s Q (χ^2^) test and quantified using the I^2^ statistic. I^2^ values of approximately 25%, 50%, and 75% were used to represent low, moderate, and high heterogeneity, respectively. An χ^2^ *p*-value < 0.10 was considered suggestive of statistically significant heterogeneity, given the limited power of the test when few studies are included. A random-effects model was used for all pooled analyses due to the anticipated clinical and methodological variability across the studies. Sensitivity analyses, including leave-one-out analyses and exclusion of studies assessed as having a high risk of bias, were prespecified to explore potential sources of heterogeneity if substantial heterogeneity was present. Subgroup analyses based on clinically relevant factors were also prespecified where sufficient data were available. Publication bias was planned to be assessed using visual inspection of funnel plots and Egger’s regression test only when at least 10 studies were available for a given outcome, as formal tests for funnel plot asymmetry are unreliable with fewer studies. The risk-of-bias evaluation revealed differences in the included studies’ methodological quality. Baojun Suo (2023) [22], showed a generally low risk of bias in the majority of domains, with only minor issues with outcome measurement. Although there were some issues with the selection of reported results, Lingyun Zhang (2019) [21], likewise demonstrated primarily low risk. Internal validity may be impacted by Zhang’s (2023) [23] low risk in a number of domains and high risk in relation to deviations from planned interventions. On the other hand, although maintaining low risk in outcome measurement, Yi Lin (2025) [20], demonstrated significant methodological limitations, including a high risk of bias in the randomization process and selective reporting. Yu Huang (2023) [13], showed a mixed profile, indicating concerns about incomplete outcome data but low risk in randomization and outcome measurement. Risk of biases is depicted in Figure 2 and Figure 3. Formal assessment of publication bias using Egger’s regression test was not performed due to the presence of fewer than 10 studies for each outcome. Additionally, due to low heterogeneity, sensitivity or subgroup analysis was not performed, and the limited number of included studies precluded meaningful exploratory analyses.

### 3.4. Meta-Analysis

This meta-analysis used Review Manager (Rev-Man version 5.4). Forest plots were generated to visually display the results. The outcomes were demonstrated as odds ratios (OR) with a 95% confidence interval using the random effects model. An I^2^ value > 50% and a *p*-value > 0.05 were considered evidence of substantial heterogeneity among studies.

**Figure 4:**
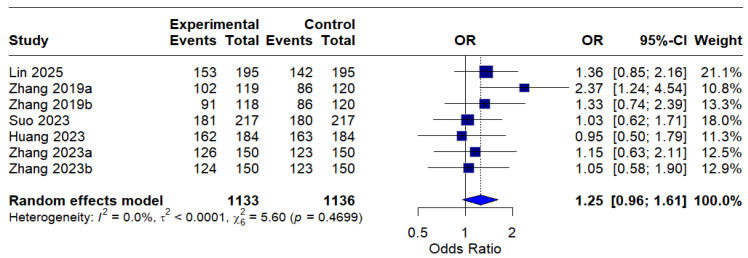
Forest Plot: Intention-to-treat (ITT) eradication rates (OR) [13,20,21,22,23]. The text in bold represents the pooled odds ratio.

The intention-to-treat (ITT) analysis indicated a pooled odds ratio (OR) of 1.25 (95% CI: 0.96–1.61), indicating a trend favoring MBQT over standard therapy in terms of eradication rates; nevertheless, this finding did not achieve statistical significance. The absence of heterogeneity (I^2^ = 0.0%) indicates consistency among studies included in the ITT analysis. This is depicted in Figure 4.

**Figure 5:**
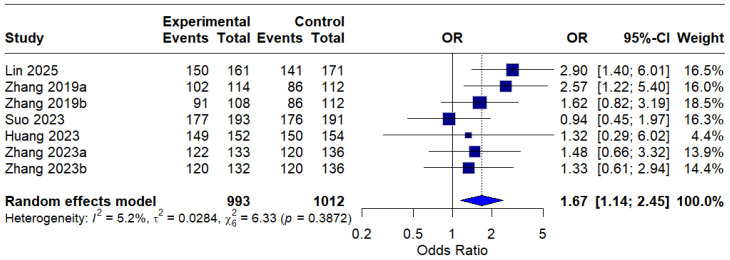
Forest Plot: Per-Protocol (PP) eradication rates OR [13,20,21,22,23]. The text in bold represents the pooled odds ratio.

In the per-protocol (PP) analysis, all seven interventions were included, and the pooled OR was 1.67 (95% CI: 1.14–2.45), indicating a statistically significant improvement in eradication rates with MBQT over standard therapy. The low heterogeneity (I^2^ = 5.2%) suggests that the outcome is reproducible across different studies. This is depicted in Figure 5.

**Figure 6:**
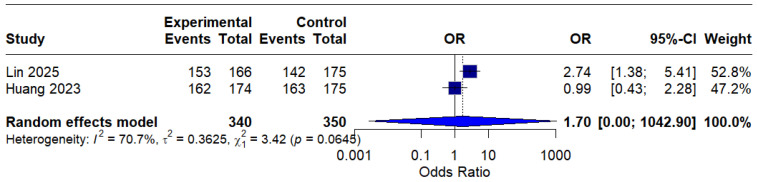
Forest Plot: Modified intention-to-treat (mITT) analysis [13,20]. The text in bold represents modified pooled odds ratio.

Based on only two studies, the modified intention-to-treat (mITT) analysis showed an OR of 1.70 (95% CI: 0.00–1042.90) of o, indicating a possible benefit of MBQT. However, the extremely wide confidence interval and high heterogeneity (I^2^ = 70.7%) rendered the result statistically insignificant and difficult to interpret clinically. This is presented in (Figure 6).

**Figure 7:**
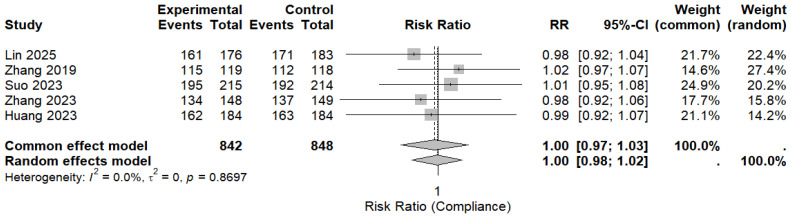
The pooled analysis for compliance rates between MBQT and standard BQT [13,20,21,22,23]. The text in bold shows the pooled RR of compliance rates.

Five randomized controlled trials reported treatment compliance and were included in the pooled analysis. Overall compliance was high in both the MBQT and comparator groups. The pooled analysis revealed no significant difference in compliance rates between MBQT and standard BQT (RR ≈ 1.00, 95% CI (0.98–1.02)), with low heterogeneity (I^2^ ≈ 0%). This is depicted in (Figure 7). These findings also indicate that MBQT does not adversely affect the treatment adherence.

**Figure 8:**
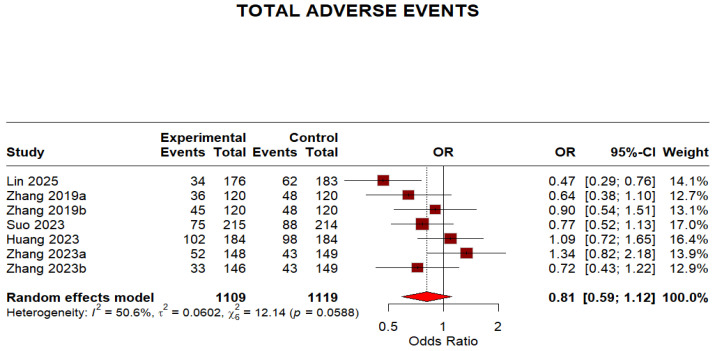
Forest plot: Adverse effects of Minocycline-Containing Bismuth Quadruple Therapies Versus Standard First-Line Bismuth Quadruple Therapies [13,20,21,22,23]. The text in bold shows the pooled OR of adverse events.

Regarding safety outcomes, the overall adverse events were not significantly different because the 95% CI crosses 1, despite a point estimate < 1 (OR 0.81, 95% CI 0.59–1.12). The moderate heterogeneity (I^2^ = 50.6%) indicates some variation in adverse event reporting across studies. This is shown in (Figure 8).

This meta-analysis shows a positive trend for the efficacy of MBQT for *H. pylori*; the most compelling evidence comes from the per-protocol analysis, which supports its use in patients who adhere to therapy.

To improve clarity for readers, eradication outcomes were additionally presented as absolute percentages. In the intention-to-treat analysis, eradication rates with minocycline-containing regimens ranged from approximately 78% to 94%, compared with ranging 73% to 94% in the control groups (Table 2). In the per-protocol analysis, eradication rates ranged from 84% to 98% in the minocycline group and from 77% to 97% in control regimens (Table 3). Overall, absolute differences in eradication rates tended to favor minocycline-based therapy; however, several studies reported comparable eradication outcomes between the two groups.

## 4. Discussion

This meta-analysis synthesized evidence from five randomized controlled trials (RCTs) comprising seven treatment arms and 2812 participants to evaluate the efficacy and safety of minocycline-containing bismuth quadruple therapy (MBQT) compared to conventional first-line bismuth quadruple therapies (BQT) in *Helicobacter pylori* eradication. These findings align with emerging evidence, such as a recent randomized controlled trial comparing minocycline-based versus tetracycline-based BQT, which demonstrated non-inferior eradication rates (intention-to-treat (ITT) analysis: 83.4% vs. 82.9% and per protocol (PP) analysis: 91.7% vs. 92.1%) [22]. In the per-protocol (PP) analysis, MBQT demonstrated significantly higher eradication efficacy than standard therapy (pooled OR: 1.67 [1.14–2.45], I^2^ = 5.2%), indicating improved outcomes among patients who completed the assigned treatment. This finding suggests that MBQT may confer an efficacy advantage under conditions of good adherence. In contrast, the intention-to-treat (ITT) analysis showed a favorable but statistically non-significant trend toward MBQT (OR: 1.25 [0.96–1.61], I^2^ = 0.0%), indicating that superiority could not be established in the overall randomized population. The discrepancy between PP and ITT findings may reflect the influence of non-adherence or loss to follow-up in real-world settings. This highlights the need for more mITT-based data in future RCTs evaluating MBQT. In terms of safety, MBQT was associated with a lower incidence of total adverse events compared to conventional regimens (OR: 0.81 [0.59–1.12]), although this difference was not statistically significant. The moderate heterogeneity (I^2^ = 50.6%) suggests variability in adverse event reporting across trials. Still, the safety profile of MBQT appears at least comparable to that of standard therapy.

Supporting these trial-based findings, a real-world study from West China Hospital of Sichuan University evaluated a 14-day minocycline/amoxicillin-based BQT in both treatment-naïve and previously treated patients. Resistance testing revealed no *H. pylori* isolates resistant to minocycline, in contrast to a 3.0% tetracycline resistance rate. High eradication rates were observed, 94.7% (ITT) and 97.3% (PP) overall, with 100% success in treatment-experienced patients comparable to, or exceeding, those reported in the meta-analysis. Taken together, the meta-analysis and the real-world study suggest that MBQT not only matches or outperforms standard BQT in controlled trial settings but may also retain high efficacy in real-world populations, potentially due to low baseline resistance, favorable pharmacokinetics, and simplified twice-daily dosing that enhances adherence. As a pilot study, however, several limitations must be acknowledged. The sample size was relatively small, and it was conducted at a single center, which may limit generalizability [24]. Minocycline offers several benefits compared to other antibiotics. Notably, *H. pylori* exhibit relatively low resistance to it. Clinical trials on minocycline-based regimens have reported resistance rates ranging from 0% to 9.1%. These results suggest that the resistance of *H. pylori* to minocycline is similar to that observed for amoxicillin, tetracycline, and cefuroxime, while being lower than that for clarithromycin, levofloxacin, and metronidazole [22,23,25,26].

MBQT was associated with a significantly higher risk of dizziness compared to conventional regimens consistent with the vestibular and neurologic adverse effects reported for tetracycline derivatives, including minocycline. In a retrospective cohort study from Beijing, China, dizziness was the most common adverse event among patients receiving a minocycline-based quadruple regimen, occurring in 9.06% of cases, followed by abdominal discomfort (6.04%), diarrhea (4.83%), and nausea (4.59%), findings that align with earlier reports [27]. Dizziness is generally attributed to minocycline-induced, reversible vestibular disturbances, which can present as vertigo, tinnitus, and ataxia, often accompanied by nausea [28]. We also examined individual adverse events frequently reported in the included studies. MBQT was associated with a significant reduction in diarrhea (OR 0.65 [0.46–0.94], I^2^ 0. Finally, minocycline’s high cost and concerns about vestibular toxicity could limit its widespread adoption without further long-term safety data.

Furthermore, the RCT by Suo et al. [22] found no statistically significant difference in the overall incidence of adverse events between the minocycline-containing regimen and the classic BQT regimen combining tetracycline and metronidazole (34% vs. 41.1%, *p* = 0.18). Most adverse events were mild to moderate in severity, with a few cases leading to treatment intolerance. For the following outcomes, the pooled estimates did not show statistically significant differences; nausea and vomiting (OR: 1.03 [0.43–2.44], I^2^ = 76.4%), anorexia (OR: 0.60 [0.17–2.15], I^2^ = 68.1%), constipation (OR: 1.08 [0.53–2.20], I^2^ = 0%), skin rash (OR: 1.09 [0.51–2.34], I^2^ = 0%), abdominal discomfort (OR = 1.12 [0.77–1.62], I^2^ = 0.4%), headache (1.01 [0.59–1.73], I^2^ = 0%), insomnia (OR: 1.34 [0.46–3.94], I^2^ = 0%), and darkened stool (OR: 0.86 [0.57–1.29], I^2^ = 0%). Notably, many had I^2^ = 0%, indicating consistent absence of difference across trials; however, nausea/vomiting and anorexia showed high heterogeneity (I^2^ > 60%), implying inconsistent results likely driven by differences in measurement, dosing, or concomitant medications.

When interpreted using Graham’s grading system for *H. pylori* eradication, which defines eradication rates of ≥90% as good and ≥95% as excellent, the results of this meta-analysis suggest that minocycline-containing bismuth quadruple therapies do not consistently achieve optimal eradication thresholds, particularly in intention-to-treat analyses. Although some per-protocol analyses reached or exceeded the 90% benchmark, ITT eradication rates—reflecting routine clinical practice-remained below this level in most included studies.

These findings indicate that while minocycline-based regimens may offer incremental improvements over standard bismuth quadruple therapy, they do not reliably elevate eradication outcomes into the excellent range. This discrepancy between PP and ITT results likely reflects challenges related to treatment adherence, regimen complexity, and real-world implementation rather than intrinsic antimicrobial efficacy alone [29].

Despite the strengths of this analysis, several limitations must be acknowledged. First, the number of included RCTs was modest, with only five studies meeting eligibility criteria, and only two contributing to the modified intention-to-treat analysis. This limited the precision of pooled estimates, as reflected in wide confidence intervals and, in the case of the mITT analysis, substantial heterogeneity. Second, although overall heterogeneity for ITT and PP analyses was low, variability was moderate to high for certain safety outcomes, particularly nausea and taste disturbance, likely reflecting differences in adverse event reporting, dosing strategies, or concomitant antibiotics used across studies. Third, most included trials were conducted in Asia, particularly China, where the prevalence of antibiotic resistance patterns, health system factors, and patient adherence may differ from Western or African settings, thus limiting the global generalizability of these findings. Fourth, none of the included trials incorporated resistance-guided therapy, which is increasingly advocated in high-resistance settings. As such, it remains unclear whether MBQT maintains its efficacy in populations with complex resistance profiles. Although the Maastricht VI/Florence consensus prioritizes susceptibility-guided therapy, it also acknowledges real-world constraints related to antibiotic availability and feasibility. In such contexts, minocycline may serve as a pragmatic alternative to tetracycline when resistance rates are low and susceptibility testing is unavailable.

Taken together, this meta-analysis supports MBQT as a viable alternative first-line regimen for *H. pylori* eradication, especially in contexts where tetracycline is inaccessible or poorly tolerated. The per-protocol superiority of MBQT suggests that, in adherent patients, the inclusion of minocycline, a second-generation tetracycline, offers several theoretical advantages. It exhibits potent antibacterial activity against *H. pylori*, better gastric tissue penetration, and a more favorable side-effect profile than tetracycline. Clinicians should remain vigilant regarding dizziness, but the overall safety profile is reassuring [17,18]. The Maastricht VI/Florence consensus, developed by an international panel of 41 experts, emphasizes the imperative to tailor *H. pylori* treatment regimens in response to evolving antibiotic resistance and constraints in drug accessibility, thereby reinforcing the rationale for considering minocycline as a viable substitute for tetracycline when necessary [6].

## 5. Conclusions

This meta-analysis indicates that minocycline-containing bismuth quadruple therapy is at least as effective and safe as conventional tetracycline-based regimens for *H. pylori* eradication and may offer superior efficacy among adherent patients. In the per-protocol analysis, MBQT demonstrated superior efficacy, as it demonstrated a statistically significant increase in eradication rates compared with standard therapy (pooled OR: 1.67; 95% CI: 1.14–2.45; I^2^ = 5.2%). Interpretation of these findings is limited by the small number of included RCTs, the reliance of the observed efficacy advantage primarily on the per-protocol analysis, and the fact that only two studies contributed to the modified intention-to-treat analysis, resulting in wide confidence intervals and substantial heterogeneity; additionally, moderate heterogeneity in safety outcomes suggests variability in adverse event reporting across studies.

## Figures and Tables

**Table 1:** Baseline characteristics of the included trials.

Study ID	Sample Size	Age (Mean ± SD)	Male (%)	BMI (Mean ± SD)	Peptic Ulcer Disease (n)	Intervention Arm	Control Arm	Duration of Therapy	Country
Lin 2025 [20]	390	43.26 ± 13.49	46.41	23.04 ± 2.83	42	Minocycline+ Ornidazole + Esomeprazole + Bismuth (MOEB)	Amoxicillin + Clarithromycin + Esomeprazole + Bismuth (ACEB)	July 2021–May 2022	China
Zhang 2019 [21]	360	42.2 ± 13.79	46.39	23.4 ± 3.85	77	Rabeprazole + Minocycline + Amoxicillin + Bismuth (RMAB group) Rabeprazole + Minocycline + Metronidazole + Bismuth (RMMB group)	Rabeprazole + Amoxicillin + Clarithromycin + Bismuth (RACB group)	June 2017–October 2018	China
Suo 2023 [22]	434	41.6 ± 12.95	49.08	23.05 ± 2.60	30	Esomeprazole + Minocycline + Metronidazole + Bismuth potassium citrate	Esomeprazole + Tetracycline + Metronidazole + Bismuth	April 2019–November 2021	China
Huang 2023 [13]	368	47.15 ± 11.50	38.04	NR	34	Esomeprazole + Minocycline + Metronidazole + Bismuth	Esomeprazole + Tetracycline + Metronidazole + Bismuth	October 2023–December 2022	China
Zhang 2023 [23]	450	40.23 ± 12.78	50.22	22.7 ± 2.34	32	Esomeprazole + Minocycline + Metronidazole + Bismuth (Min-Met BQT) Esomeprazole + Minocycline + Cefuroxime + Bismuth (Min-Cef BQT)	Esomeprazole + Cefuroxime + Metronidazole + Bismuth (Cef-Met BQT)	March 2019–June 2022	China

**Table 2:** Data are shown as n/N (%) based on the intention-to-treat population. Absolute difference indicates the percentage difference between minocycline-containing and control regimens.

Study	Minocycline (n/N)	Eradication %	Control (n/N)	Eradication %	Absolute Difference (%)
Lin 2025 [20]	150/168	89.3%	141/176	80.1%	+9.2%
Zhang 2019 (Arm 1) [21]	102/120	85.0%	86/118	72.9%	+12.1%
Zhang 2019 (Arm 2) [21]	91/116	78.4%	86/118	72.9%	+5.5%
Suo 2023 [13]	177/201	88.1%	176/198	88.9%	−0.8%
Huang 2023 [23]	149/158	94.3%	150/160	93.8%	+0.5%
Zhang 2023 (Arm 1) [23]	122/140	87.1%	120/145	82.8%	+4.3%
Zhang 2023 (Arm 2) [23]	120/138	87.0%	120/145	82.8%	+4.2%

Eradication Rates Expressed as Percentages. Eradication Rates of Intention-to-Treat (ITT) Analysis.

**Table 3:** Data are shown as n/N (%) based on the per-protocol population. Absolute difference indicates the percentage difference between minocycline-containing and control regimens.

Study	Minocycline (n/N)	Eradication %	Control (n/N)	Eradication %	Absolute Difference (%)
Lin 2025 [20]	150/161	93.2%	141/171	82.5%	+10.7%
Zhang 2019 (Arm 1) [21]	102/114	89.5%	86/112	76.8%	+12.7%
Zhang 2019 (Arm 2) [21]	91/108	84.3%	86/112	76.8%	+7.5%
Suo 2023 [22]	177/193	91.7%	176/191	92.1%	−0.4%
Huang 2023 [13]	149/152	98.0%	150/154	97.4%	+0.6%
Zhang 2023 (Arm 1) [23]	122/133	91.7%	120/136	88.2%	+3.5%
Zhang 2023 (Arm 2) [23]	120/132	90.9%	120/136	88.2%	+2.7%

Eradication Rates of Per-Protocol (PP) Analysis.

## Data Availability

All data supporting the findings are included within this article.

## Supplementary Materials

The following supporting information can be downloaded at: https://www.mdpi.com/article/10.3390/idr18010016/s1.

## Abbreviations

All abbreviations have been outlined at the first mention of the word but briefly:

MBQT	Minocycline-containing bismuth quadruple therapy
RCT	Randomized controlled trial
OR	Odds ratio
ITT	Intention to treat analysis

## Appendix A

**Database**: PubMed (MEDLINE)
**Search Date**: 28 May 2025 **Results**: 1080 records
Search Strategy:
(“Helicobacter pylori”[MeSH Terms] OR “Helicobacter Infections”[MeSH Terms] OR “Campylobacter pylori”[Title/Abstract] OR “Helicobacter pylori”[Title/Abstract] OR “h pylori”[Title/Abstract] OR “h pylori”[Title/Abstract]) AND (“Minocycline”[MeSH Terms] OR “Minocycline”[Title/Abstract] OR “Minocycline-Containing”[Title/Abstract] OR ((“Bismuth”[MeSH Terms] OR “Bismuth”[Title/Abstract]) AND (“quadruple therap*”[Title/Abstract] OR “quadruple regimen*”[Title/Abstract])))
**Database**: **Cochrane CENTRAL**
**Search Date**: 28 May 2025 **Results**: 44 records
Search Strategy:
#1 MeSH descriptor: [Helicobacter Infections] this term only 2734
#2 MeSH descriptor: [Helicobacter pylori] this term only 2569
#3 (“Helicobacter pylori”):ti,ab,kw 6213
#4 (“Helicobacter pylori infection”):ti,ab,kw 2158
#5 #1 OR #2 OR #3 OR #4 6275
#6 MeSH descriptor: [Minocycline] this term only 677
#7 (“minocycline”):ti,ab,kw 1348
#8 #6 OR #7 1348
#9 #5 AND #8 44
**Database**: **Scopus**
**Search Date**: 28 May 2025 **Results**: 384
Search Strategy:
TITLE-ABS-KEY(“Helicobacter pylori” OR “Helicobacter Infection*” OR “Campylobacter pylori” OR “H. pylori” OR “h pylori”))
AND
(TITLE-ABS-KEY((Bismuth AND (“quadruple therap*” OR “quadruple regimen*”)) OR Minocycline OR “Minocycline-containing”))
**Database**: **Embase by Ovid**
**Search Date**: 28 May 2025 **Results**: 130
Search Strategy:
(“Helicobacter pylori” OR “Helicobacter Infection*” OR “Campylobacter pylori” OR “H. pylori” OR “h pylori”)
AND
((“Bismuth” AND (“quadruple therap*” OR “quadruple regimen*”) OR (“Minocycline” OR “Minocycline-Containing”))
**Database**: **ScienceDirect**
**Search Date**: 28 May 2025 **Results**: 194
Search Strategy:
Title, abstract, keywords: (“Helicobacter pylori” OR “Helicobacter Infection” OR “H. pylori” OR “h pylori”) AND ((“Bismuth” AND (“quadruple therapy” OR “quadruple regimen”) OR (“Minocycline” OR “Minocycline-Containing”))

## References

[1] Li Y., Choi H., Leung K., Jiang F., Graham D.Y., Leung W.K. (2023). Global prevalence of Helicobacter pylori infection between 1980 and 2022: A systematic review and meta-analysis. Lancet Gastroenterol. Hepatol..

[2] Kusters J.G., van Vliet A.H.M., Kuipers E.J. (2006). Pathogenesis of *Helicobacter pylori* Infection. Clin. Microbiol. Rev..

[3] Møller H., Heseltine E., Vainio H. (1995). Working group report on schistosomes, liver flukes and *Helicobacter pylori*. Meeting held at IARC, LYON, 7–14 June 1994. Int. J. Cancer.

[4] Sugano K., Tack J., Kuipers E.J., Graham D.Y., El-Omar E.M., Miura S., Haruma K., Asaka M., Uemura N., Malfertheiner P. (2015). Kyoto global consensus report on *Helicobacter pylorigastritis*. Gut.

[5] Savoldi A., Carrara E., Graham D.Y., Conti M., Tacconelli E. (2018). Prevalence of Antibiotic Resistance in Helicobacter pylori: A Systematic Review and Meta-analysis in World Health Organization Regions. Gastroenterology.

[6] Malfertheiner P., Megraud F., Rokkas T., Gisbert J.P., Liou J.M., Schulz C., Gasbarrini A., Hunt R.H., Leja M., El-Omar E.M. (2022). Management of Helicobacter pylori infection: The Maastricht VI/Florence consensus report. Gut.

[7] Chey W.D., Leontiadis G.I., Howden C.W., Moss S.F. (2017). ACG Clinical Guideline: Treatment of Helicobacter pylori Infection. Am. J. Gastroenterol..

[8] Nazarian S., Akhondi H. (2025). Minocycline. StatPearls.

[9] Nyssen O.P., Vaira D., Tepes B., Kupcinskas L., Bordin D., Pérez-Aisa Á., Gasbarrini A., Castro-Fernández M., Bujanda L., Garre A. (2021). Room for Improvement in the Treatment of Helicobacter pylori Infection. J. Clin. Gastroenterol..

[10] Siavoshi F., Sahraee M., Ebrahimi H., Sarrafnejad A., Saniee P. (2018). Natural fruits, flowers, honey, and honeybees harborHelicobacter pylori-positive yeasts. Helicobacter.

[11] Thung I., Aramin H., Vavinskaya V., Gupta S., Park J.Y., Crowe S.E., Valasek M.A. (2015). Review article: The global emergence of Helicobacter pylori antibiotic resistance. Aliment. Pharmacol. Ther..

[12] Huang Y., Qiu S., Guo Y., Chen J., Li M., Ding Z., Zhang W., Liang X., Lu H. (2024). Optimization of Minocycline-Containing Bismuth Quadruple Therapy for Helicobacter pylori Rescue Treatment: A Real-World Evidence Study. Helicobacter.

[13] Huang Y., Chen J., Ding Z., Chen X., Liang X., Zeng X., Xu F., Han Y., Lu H. (2023). Minocycline vs. tetracycline in bismuth-containing quadruple therapy for Helicobacter pylori rescue treatment: A multicentre, randomized controlled trial. J. Gastroenterol..

[14] Zhou K., Li C.L., Zhang H., Suo B.J., Zhang Y.X., Ren X.L., Wang Y.-X., Mi C.-M., Ma L.-L., Zhou L.-Y. (2024). Minocycline in the eradication of Helicobacter pylori infection: A systematic review and meta-analysis. World J. Gastroenterol..

[15] Kim S.Y., Chung J.W. (2020). Best Helicobacter pylori Eradication Strategy in the Era of Antibiotic Resistance. Antibiotics.

[16] Ding Y., Zuo X., Li Y. (2021). Systematic Review and Meta-Analysis of 10 and 14 Days Bismuth-Containing Quadruple Therapy for Helicobacter Pylori Eradication.

[17] Ierardi E., Losurdo G., Fortezza R.F.L., Principi M., Barone M., Leo A.D. (2019). Optimizing proton pump inhibitors in Helicobacter pylori treatment: Old and new tricks to improve effectiveness. World J. Gastroenterol..

[18] Page M.J., McKenzie J.E., Bossuyt P.M., Boutron I., Hoffmann T.C., Mulrow C.D., Shamseer L., Tetzlaff J.M., Akl E.A., Brennan S.E. (2021). The PRISMA 2020 statement: An updated guideline for reporting systematic reviews. BMJ.

[19] Zhang L., Zhou L., Song Z., Ding Y., Bai P. (2015). Minocycline quadruple versus tailored therapy in retreatment of Helicobacter pylori infection. Zhonghua Nei Ke Za Zhi.

[20] Lin Y., Lin X., Suo B., Chen Q., Cheng X., Lin Z., Huang X. (2025). Randomized multicenter trial comparing minocycline and ornidazole with classical quadruple therapy in *Helicobacter pylori* treatment. Sci. Rep..

[21] Zhang L., Lan Y., Wang Q., Zhang Y., Si X. (2019). Application of Minocycline-Containing Bismuth Quadruple Therapies as First-Line Regimens in the Treatment of Helicobacter pylori. Gastroenterol. Res. Pract..

[22] Suo B., Tian X., Zhang H., Lu H., Li C., Zhang Y., Ren X., Yao X., Zhou L., Song Z. (2023). Bismuth, esomeprazole, metronidazole, and minocycline or tetracycline as a first-line regimen for Helicobacter pylori eradication: A randomized controlled trial. Chin. Med. J..

[23] Zhang Y., Suo B., Tian X., Zhang H., Lu H., Yao X., Li C., Ren X., Zhou L., Song Z. (2023). New regimens as first-line eradication therapy for Helicobacter pylori infection in patients allergic to penicillin: A randomized controlled trial. Helicobacter.

[24] You S., Tang X., Zhou J., Shen Y., Song X., Benghezal M., Marshall B.J., Tang H., Li H. (2024). Minocycline/Amoxicillin-Based Bismuth Quadruple Therapy for Helicobacter pylori Eradication: A Pilot Study. Microorganisms.

[25] Barza M., Schiefe R.T. (1977). Antimicrobial spectrum, pharmacology and therapeutic use of antibiotics, Part 1: Tetracyclines. Am. J. Health Syst. Pharm..

[26] Song Z., Suo B., Zhang L., Zhou L. (2016). Rabeprazole, Minocycline, Amoxicillin, and Bismuth as First-Line and Second-Line Regimens for Helicobacter pylori Eradication. Helicobacter.

[27] Si X.B., Zhang L.Y., Yang S., Chen X.L., Shi Y.Y., Lan Y., Ding S.-G. (2024). The Efficacy and Safety of Minocycline-Containing Quadruple Therapies Against Helicobacter pylori Infection: A Retrospective Cohort Study. Infect. Drug Resist..

[28] Martins A.M., Marto J.M., Johnson J.L., Graber E.M. (2021). A Review of Systemic Minocycline Side Effects and Topical Minocycline as a Safer Alternative for Treating Acne and Rosacea. Antibiotics.

[29] Graham D.Y., Lu H., Yamaoka Y. (2007). A report card to grade Helicobacter pylori therapy. Helicobacter.

